# Simplifying Busulfan Conditioning in Children: Once Daily vs. Four-Times-Daily IV Dosing in Hematopoietic Cell Transplantation

**DOI:** 10.3390/jcm15218754

**Published:** 2026-11-09

**Authors:** Manon Dzedzej, Charlotte Nazon, Guillaume Becker, Amelia-Naomi Sabo, Véronique Kemmel, Catherine Paillard

**Affiliations:** 1 Service d’Hématologie et d’Oncologie Pédiatrique, Hôpitaux Universitaires de Strasbourg, 67200 Strasbourg, Francecatherine.paillard@chru-strasbourg.fr (C.P.); 2 Service Pharmacie, Hôpitaux Universitaires de Strasbourg, 67200 Strasbourg, France; 3 Laboratoire de Pharmacologie et Toxicologie Neurocardiovasculaire, Unité de Recherche 7296, Faculté de Médecine de Maïeutique et des Sciences de la Santé, Centre de Recherche en Biomédecine de Strasbourg (CRBS), Université de Strasbourg, 67085 Strasbourg, France; amelia-naomi.sabo@chru-strasbourg.fr (A.-N.S.); veronique.kemmel@chru-strasbourg.fr (V.K.); 4 Laboratoire de Biochimie et Biologie Moléculaire, Secteur de Chromatographie et Spectrométrie de Masse, Hôpitaux Universitaires de Strasbourg, 67098 Strasbourg, France; 5 Laboratoire d’ImmunoRhumatologie Moléculaire, INSERM UMR_S 1109, LabEx Transplantex, Fédération de Médecine Translationnelle de Strasbourg, Université de Strasbourg, 67085 Strasbourg Cedex, France

**Keywords:** hematopoietic cell transplantation, conditioning regimen, sinusoidal obstruction syndrome

## Abstract

**Background/Objectives**: Busulfan is a key component of conditioning regimens for hematopoietic cell transplantation (HCT). Although once-daily (OD) intravenous administration is logistically advantageous over the conventional four-times-daily (QID) schedule, concerns remain regarding a potential increased risk of hepatic veno-occlusive disease (VOD or sinusoidal obstruction syndrome/SOS), particularly in children. **Methods**: We retrospectively reviewed all pediatric patients receiving busulfan-based conditioning with therapeutic drug monitoring (TDM) at a single center (January 2015–December 2024). The primary analysis compared VOD/SOS incidence within 100 days post-HCT, diagnosed by revised EBMT criteria, between the OD and allogeneic QID groups (QIDallo). Secondary analyses evaluated VOD/SOS severity, busulfan pharmacokinetic (PK) parameters, and clinical risk factors and compared the OD and total QID groups. **Results**: One hundred HCT procedures were included (36 OD, 64 QIDtot including 39 QIDallo). There was no statistically significant between-group difference (*p* = 0.777 for OD/QIDallo and *p* = 0.960 for OD/QIDtot), including after adjustment for age and disease. OD yielded higher C_max_ than QIDallo and QID (*p* < 0.001), but PK parameters were not associated with VOD/SOS. Independent risk factors were age ≤ 5 years (*p* = 0.001) and thalassemia (*p* = 0.035). **Conclusions**: In this pediatric cohort, PK-guided OD busulfan was not associated with an increased VOD/SOS risk despite higher C_max_, supporting its use in children when TDM is implemented. Young age and thalassemia remained the principal determinants of VOD/SOS.

## 1. Introduction

Busulfan, an alkylating agent with strong myeloablative properties, is a cornerstone of many conditioning regimens for hematopoietic cell transplant (HCT). First used in HCT conditioning in oral form in the 1980s, its intravenous form, developed in the 2000s, significantly reduced inter-individual variability in drug exposure [1,2]. Nevertheless, busulfan has a narrow therapeutic index, with underexposure increasing the risk of graft failure and relapse, while overexposure heightens the likelihood of severe toxicities [3,4,5]. Therefore, therapeutic drug monitoring (TDM) and careful pharmacokinetic (PK)-guided dosing have become standard practice for at-risk patients in many centers. 

Notably, busulfan-based conditioning regimens have been linked with increased risk of veno-occlusive disease (VOD, also known as sinusoidal obstruction syndrome, SOS). VOD/SOS is a severe complication in HCT, particularly in children, with an incidence of approximately 20%, but up to 50 to 60% in high-risk individuals [6]. It is due to multifactorial endothelial damage and has a significant impact on early survival [7,8,9]. Reducing the burden of VOD/SOS is therefore a priority in pediatric HCT.

Traditionally, busulfan has been administered in four daily doses every six hours (QID). This schedule was inherited from oral regimens and was aimed at mitigating variability in bioavailability [2]. However, the advent of intravenous formulations has made it possible to explore alternative dosing schedules. Once-daily administration (OD) was introduced as a more convenient option, offering potential advantages such as simplified nursing procedures, improved patient comfort, and reduced hospital costs [2,10,11,12,13]. PK studies have suggested that OD dosing can achieve systemic exposures equivalent to QID regimens in terms of total area under the curve (AUC) [10,11,12]. However, equivalence in AUC does not necessarily imply equivalence in toxicity. OD administration results in a higher single-dose maximum plasma concentration (C_max_), which could be a risk factor for endothelial injury and VOD/SOS, as suggested by recent studies [14]. 

The current literature includes multiple studies comparing OD and QID busulfan administration, but pediatric-specific data remain scarce [15]. Most available studies suggest equivalence in efficacy, yet evidence on safety is conflicting, and concerns regarding VOD/SOS persist [14,15,16]. Given the clinical significance of this complication and its disproportionate impact in children compared to adults, further research focusing on pediatric populations is needed.

The aim of this study was to compare the incidence of VOD/SOS according to busulfan administration schedule in a cohort of pediatric patients undergoing HCT in a single tertiary center. By focusing on this high-risk population, we sought to clarify whether OD administration, despite its logistical advantages, carries a higher risk of hepatic toxicity compared to the conventional QID regimen in children.

## 2. Materials and Methods

We retrospectively included all consecutive patients who underwent HCT (autologous or allogeneic) in the Department of Pediatric Hematology and Oncology (Strasbourg University Hospital, Strasbourg, France) between January 2015 and December 2024. Inclusion criteria were age < 25 years at the time of HCT, intravenous busulfan as part of the conditioning regimen, administered either OD or QID, and busulfan dose adjustment based on PK monitoring of the AUC. Exclusion criteria were the use of oral busulfan and an incomplete busulfan course or missing PK data.

The primary analysis was the comparison of VOD/SOS occurrence within 100 days post-HCT, a window chosen to capture late-onset cases while covering typical onset (usually ≤21 days, rarely >50 days), between the OD and allogeneic QID groups. Secondary analyses assessed VOD/SOS severity grade, its association with busulfan PK parameters (C_max_, daily AUC, cumulative AUC [AUC_cum_]), and potential risk factors (demographics, underlying disease, pre-transplant hepatic status, conditioning regimen, transplant characteristics). VOD/SOS incidence was also compared between OD and the entire QID cohort. C_max_ was defined as the maximum plasma concentration observed across all measured pharmacokinetic assessments (i.e., across all treatment days with PK sampling).

VOD/SOS was retrospectively assessed using revised European Society for Blood and Marrow Transplantation (EBMT) criteria (ultrasound findings other than hepatomegaly/ascites excluded per EBMT recommendations) and graded accordingly [6]. Each patient underwent a pre-transplant abdominal ultrasound, performed by a trained radiologist, which provided an individual baseline for liver size and ascites. Hepatomegaly and ascites were therefore defined as an increase relative to each patient’s own baseline. For bilirubin, the EBMT criterion was applied as a rise from baseline on 3 consecutive days or a value ≥2 mg/dL (34 µmol/L) within 72 h; a stable elevated bilirubin related to chronic hemolysis was therefore not counted. For comparison with the prior literature, incidence was also assessed using modified Seattle and Baltimore criteria [17,18].

Demographic and disease-related data (age, sex, height, weight, body surface area, pre-existing liver conditions, underlying disease, disease status at HCT, prior hepatotoxic therapies) were extracted from electronic and paper records. Conditioning data, including busulfan schedule (OD vs. QID), associated agents, anti-thymocyte globulin (ATG) use, graft source, donor, and transplant type (autologous/allogeneic), were retrieved from records and the institutional ChimioWeb^®^ software v6.0.

Busulfan TDM followed Strasbourg University Hospital’s standard procedure. Busulfan was administered as a 3 h intravenous infusion for the OD schedule and a 2 h infusion for the QID schedule. The starting dose was calculated based on patient body weight, according to institutional protocol (Table S1). No test dose was administered prior to the first therapeutic dose. For OD regimens, sampling occurred at doses 1–4 (pre-infusion and 3.5, 4, 6, 7, 9 h after the start of the infusion); for QID, at doses 1, 6, 10, and 14 (pre-dose and 2.5, 3, 4, 5 h after the start of the infusion). Samples were collected in heparinized plasma tubes (with or without separating gel), centrifuged, and stored at −20 °C until analysis. Busulfan concentrations were measured in plasma using an in-house LC-MS/MS method, accredited under ISO 15189 by the French Accreditation Committee (COFRAC) [19]. Busulfan quantification was performed on an HPLC-Shimadzu system coupled to a SCIEX Triple Quad 5500 mass spectrometer (Kinetex^®^ C8 column, 100 × 3.0 mm, 2.6 µm, Phenomenex, Torrance, CA, USA). The limit of detection (LOD) was 20 µg/L, the limit of quantification (LOQ) was 50 µg/L, and the upper limit of linearity was 5000 µg/L. AUC was calculated by the linear trapezoidal method (GraphPad Prism^®^) to guide dose adjustment (adjusted dose = administered dose × target AUC/observed AUC). For the OD schedule, a full pharmacokinetic profile was obtained at each of the four daily administrations (one profile per treatment day), and the AUC was calculated for each dose/day using the linear trapezoidal method. The daily dose was adjusted to target an AUC(0–24) of 5200 µM.min, equivalent to 21,320 µg.h/L. For the QID schedule, a full pharmacokinetic profile was obtained for only one of the four daily doses per treatment day (doses 1, 6, 10, and 14, corresponding to one PK assessment per day), as intensive sampling at all four doses on each day was not feasible in routine practice. The AUC measured for this single dose was used to estimate exposure for the remaining three doses administered that day, assuming comparable exposure per dose within the same day. The target AUC(0–6) was 1300 µM.min, equivalent to a daily cumulative exposure of 5200 µM.min, allowing comparison with the OD target on the same daily basis. Cumulative AUC (AUC_cum_) values are reported in mg.h/L, consistent with the units conventionally used in the busulfan pharmacokinetic literature, whereas single-day/single-dose AUC values used for dose adjustment were expressed in µM.min. For clarity and consistency with the AUC_cum_ values reported in mass units (mg.h/L), the daily AUC target can be converted as follows: 5200 µM.min corresponds to approximately to 21,329 µg.h/L or 21.3 mg.h/L (using busulfan’s molar mass of 246.1 g/mol), and the per-dose QID target of 1300 µM.min corresponds to approximately 5332 µg.h/L or 5.3 mg.h/L. Over the four treatment days, this daily target yields a cumulative AUC of approximately 85.2 mg.h/L. The AUC_cum_ therapeutic window was 78–101 mg.h/L per the literature, with an internal target of 85–95 mg.h/L. Dose adjustments were made jointly by hematology and biochemistry teams after each assessment. 

Statistical analyses used Jamovi (v2.6.44). To explore temporal changes in supportive care and diagnostic practice, the optimal transplantation-date threshold was identified by ROC analysis (Youden index), and VOD/SOS incidence before and after this threshold was compared using Fisher’s exact test. Qualitative variables were compared by χ^2^ test; quantitative variables by unpaired Student’s *t*-test. Associations with VOD/SOS were estimated as odds ratios (95% confidence interval, CI), with significant univariate variables entered into multivariate logistic regression. Significance was set at *p* < 0.05.

This retrospective study followed the Declaration of Helsinki and was approved by the Strasbourg University Hospital institutional review board. Data were anonymized, and individual consent was obtained from all subjects involved in the study.

## 3. Results

### 3.1. Study Population

A total of 100 transplants were included, corresponding to 99 patients (one patient received two transplants with busulfan-based conditioning regimens). Of these 100 grafts, 75 were allogeneic, and 25 were autologous HCT. Median age at transplant was 6.8 years (range 5 months–20.6 years); 56 recipients were male and 44 female (sex ratio 1.27). Baseline characteristics of patients are summarized in Table 1. Because all autografts used the QID schedule, we present a separate allogeneic QID subgroup (QIDallo) to enable relevant comparisons with the OD group, in addition to the comparison with the whole QID group (QIDtot).

No significant differences were observed in sex distribution between OD and QIDtot or OD and QIDallo. The OD group had a median age of 5.7 years, the QIDtot group, 7.0 years (*p* = 0.9), and the QIDallo group, 8.2 years (*p* = 0.555). Children of 5 years or less were more frequent in the OD group (20/36, 55.5%) than in the QIDallo group (11/39, 28.2%; *p* = 0.0163).

Regarding transplant indications, solid tumors all received autografts with a conditioning regimen consisting of four daily doses of busulfan (only present in the QIDtot group). Patients receiving allogeneic HCT for thalassemia all received a conditioning regimen with four daily doses of busulfan (only present in the QIDtot and QIDallo groups). Aplastic anemia and myelodysplastic syndromes (MDS) were all in the OD group. Acute myeloid leukemia (AML) was more frequent in the OD group (36.1%) than in the QIDallo (5.7%; *p* = 0.001) group. Acute lymphoblastic leukemia (ALL) was more frequent in the QIDallo (20.1%) group than in the OD group (5.1%), although this difference did not reach statistical significance (*p* = 0.089).

Ten patients with AML received gemtuzumab ozogamicin, all of whom were in the OD group (representing 27.8% of the OD group). No patients in our study received pre-HCT inotuzumab ozogamicin or actinomycin D.

### 3.2. Transplant and Donor Characteristics

Allogeneic transplant characteristics are summarized in Table 2. All 25 autografts were conditioned with QID busulfan and melphalan (Bu/Mel).

All patients received ursodeoxycholic acid as VOD/SOS prophylaxis. Defibrotide was used prophylactically in 10 patients (5 in each group) and therapeutically in all patients with diagnosed VOD/SOS.

There were no significant differences between the QIDallo and OD groups regarding graft source and ATG use. There were between-group differences regarding donor type: Matched Unrelated Donor (MUD) was more common in the OD group (41.7%) than in the QIDallo group (25.6%; *p* = 0.035); and conditioning regimens: busulfan/cyclophosphamide (Bu/Cy) was more frequent, although not significantly, in the OD group (63.9%) than in the QIDallo (41%; *p* = 0.065) group.

### 3.3. Pre-Transplant Liver Status

Table 2 summarizes pre-transplant hepatic findings. The prevalence of pre-graft liver abnormalities (hepatomegaly alone or other abnormalities) did not differ significantly between groups (*p* = 0.836 for OD/QIDtot and *p* = 0.647 for OD/QIDallo). The percentage of patients with hepatic abnormalities other than isolated hepatomegaly was 19.4% (7/36) in the OD group (asymptomatic biliary lithiasis n = 3; abnormal liver tests with ASAT/ALAT >2.5N n = 3; hilar lymphadenopathy n = 1) and 21.9% (14/64) in QIDtot (iron overload on MRI n = 6; hepatic metastases n = 2; prior VOD/SOS n = 1; hepatitis C n = 1; abnormal liver tests n = 1; hilar lymphadenopathy n = 1; increased portal flow on Doppler n = 1; steatosis n = 1), including 9 belonging to the QIDallo group (9/39; 23.1%). Pre-HCT bilirubin > 26 μmol/L was more frequent in the QIDallo group (30.8%; 12/39) than in the OD group (2.7%; 1/36, *p* = 0.002). No difference was observed when comparing the OD and QIDtot groups. Pre-transplant ferritin levels did not differ significantly across groups (*p* = 0.220 for OD/QIDtot and *p* = 0.080 for OD/QIDallo).

### 3.4. Incidence of Hepatic VOD/SOS

Within 100 days post-transplant, based on EBMT criteria, 42 clinical VOD/SOS events occurred, yielding an overall incidence of 42%: 30 in patients receiving allogeneic HCT (40%) and 12 in patients receiving autologous HCT (48%). Median time to VOD/SOS was 11 days (range 4–55 days). By EBMT criteria, 13 patients (31.0%) had Grade 1 VOD/SOS, 6 (14.3%) Grade 2, 15 (35.7%) Grade 3, and 8 (19.0%) Grade 4.

Among Grade 3 cases, classification drivers included isolated refractory thrombocytopenia > 7 days (n = 7), isolated hyperbilirubinemia > 34 μmol/L (n = 3), refractory thrombocytopenia and coagulation impairment not requiring replacement therapy (n = 2), hyperbilirubinemia and coagulation impairment not requiring replacement therapy (n = 1), elevated liver enzymes > 2–5ULN with refractory thrombocytopenia (n = 1), and elevated liver enzymes > 2–5ULN with clinically significant ascites requiring paracentesis (n = 1).

All patients with Grade 4 VOD/SOS (n = 8) met EBMT multi-organ criteria. Four patients died in the context of suspected clinical VOD/SOS, evenly split between busulfan schedules. Among the two who had received OD busulfan, one death was considered unlikely to be attributable to VOD/SOS (septic shock at day 10, following early-onset VOD/SOS from day 4), and one followed VOD/SOS with pulmonary fluid overload and coagulopathy leading to fatal alveolar hemorrhage after allogeneic HCT. Among the two who had received QID busulfan, one followed late-onset VOD/SOS (day 55) with massive ascites and multiorgan failure after autologous HCT for neuroblastoma, and one occurred after VOD/SOS with acute kidney injury requiring renal replacement therapy after allogeneic HCT.

The prevalence of the five EBMT diagnostic components in VOD/SOS cases was: refractory thrombocytopenia 88% (37/42), weight gain 86% (36/42), hepatomegaly 93% (39/42), ascites 64% (27/42), and bilirubin increase 71% (30/42).

To enable comparison with the prior literature, we also applied modified Seattle and Baltimore definitions. Overall VOD/SOS incidence was 26% by modified Seattle criteria and 8% by Baltimore criteria.

To investigate temporal changes in supportive care and the incidence of VOD/SOS, the optimal cut-off was 30 July 2019 (AUC = 0.604, 95% CI 0.491–0.715; *p* = 0.078 for the AUC versus 0.5; sensitivity = 64.3%, specificity = 60.3%). VOD/SOS occurred in 15/50 patients (30.0%) transplanted before this date and in 27/50 patients (54.0%) transplanted on or after it (Fisher’s exact test, *p* = 0.025). 

### 3.5. Comparison of Busulfan Dosing Schedule

By EBMT criteria, VOD/SOS incidence was 41.7% in the OD (15/36), 38.5% (15/39) in QIDallo, and 42.2% in QIDtot (27/64) groups. There was no statistically significant between-group difference (*p* = 0.777 for OD/QIDallo and *p* = 0.960 for OD/QIDtot).

VOD/SOS severity distributions were similar across groups (Table S2).

Crude odds ratios (OR) for VOD/SOS were OD vs. QIDallo: OR 1.14 (95% CI 0.453–2.88; *p* = 0.777) and OD vs. QID: OR 0.979 (95% CI 0.428–2.24; *p* = 0.960). After adjustment for age and underlying disease, the schedule remained non-significant (adjusted OR for OD vs. QIDallo 1.32, 95% CI 0.4305–4.06; *p* = 0.626; adjusted OR for OD vs. QIDtot 1.31, 95% CI 0.4209–4.08; *p* = 0.640).

### 3.6. VOD/SOS Risk Factors

We examined clinical (age, sex, year of transplant, underlying disease, pre-graft liver abnormalities, transplant type, donor type, conditioning), biological (ferritin, bilirubin, recipient CMV status), and PK variables (C_max_, AUC_max_, AUC_cum_).

Three variables were significantly associated with VOD/SOS: age ≤ 5 years: OR 4.44 (95% CI 1.90–10.4, *p* < 0.001), abnormal pre-graft ferritin: OR 3.55 (95% CI 1.08–11.7, *p* = 0.039), and thalassemia: OR 11.4 (95% CI 1.35–96.6, *p* = 0.026).

Sex, transplant year, pre-graft liver abnormalities (overall), transplant type (autologous vs. allogeneic), donor type, conditioning regimen, pre-graft bilirubin, recipient CMV status, and busulfan PK parameters (C_max_, AUC_max_, AUC_cum_) were not significantly associated with VOD/SOS. After adjusting for major risk factors, only age and thalassemia remained independently associated with VOD/SOS: adjusted OR for age ≤ 5 years 4.65 (95% CI 1.82–11.88, *p* = 0.001) and for thalassemia 10.91 (95% CI 1.18–101.27, *p* = 0.035). Pre-graft ferritin lost significance after adjustment.

### 3.7. Busulfan Pharmacokinetics and VOD/SOS

C_max_ did not differ between patients with and without VOD/SOS (overall mean 2676 vs. 2538 µg/L; *p* = 0.599). Within schedules, C_max_ was not associated with VOD/SOS (OD: 4064 vs. 3740 µg/L; *p* = 0.295; QIDtot: 1635 vs. 1783 µg/L; *p* = 0.239).

AUC_max_ (highest daily exposure over the 4 treatment days) was not associated with VOD/SOS (overall mean 28,066 vs. 28,799 μg.h/L; *p* = 0.489). Within schedules, no significant differences were observed for OD 29,578 vs. 28,467 μg.h/L (*p* = 0.571) or QIDtot 26,931 vs. 29,008 μg·h/L (*p* = 0.082).

Exceeding the target cumulative exposure (AUC_cum_) was not linked to higher VOD risk (OR 1.44, 95% CI 0.276–7.56; *p* = 0.662), with similar findings within OD and QIDtot.

As expected, OD produced higher single-dose peaks: mean C_max_ 3903 µg/L in OD vs. 1850 µg/L in QIDtot (and 1910 µg/L in QIDallo; both *p* < 0.001). Despite these peak differences, AUC-based exposures were comparable: mean AUC_max_ 29,129 μg.h/L (OD), 28,122 μg.h/L (QIDtot; *p* = 0.355), and 27,050 μg.h/L (QIDallo; *p* = 0.077). The mean cumulative busulfan AUC was significantly higher in the OD group than in the overall QID group (89.69 vs. 84.22; *p* = 0.015). This difference was also significant when the OD group was compared with the QID allogeneic subgroup (89.69 vs. 81.66; *p* = 0.002). The distribution across below-target, on-target, and above-target categories did not differ significantly between the OD and QIDtot. However, compared with QIDallo, below-target AUC_cum_ was less frequent in OD (*p* = 0.038) (Table S3). 

## 4. Discussion

In this single-center cohort of pediatric and adolescent/young adult HCT recipients, VOD/SOS occurred in 42% of patients according to EBMT criteria (26% by modified Seattle criteria; 8% by Baltimore criteria), typically within two weeks post-HCT. As in prior pediatric reports, approximately 30% of cases in our series were anicteric, again underlining the importance of pediatric-adapted definitions [20]. The OD schedule yielded higher C_max_ and higher AUC_Cum_ than QID, with fewer patients below target compared with the QIDtot/QIDallo schedule, although mean exposure remained within the therapeutic window; neither schedule nor busulfan PK parameters (C_max_, AUC_max_, AUC_cum_) were significantly associated with VOD/SOS after adjustment. Young age (≤5 years) and thalassemia emerged as factors independently associated with VOD/SOS. 

The 42% VOD/SOS incidence in our study population appears high at first glance. In children, post-HCT VOD/SOS rates of approximately 20% are commonly reported, rising to 50–60% in high-risk settings [6]. Several risk factors for VOD/SOS were present in our population and may have contributed to this elevated incidence: busulfan-based conditioning in all patients, a very young age distribution (approximately half of the patients were aged 5 years or younger at the time of transplantation), and a high prevalence of high-risk underlying diseases (e.g., neuroblastoma 21%, thalassemia 8%). VOD/SOS incidence was higher after 2019 (54.0% vs. 30.0%). This data-driven threshold should be interpreted cautiously, but changes in patient mix, conditioning regimens, and diagnostic practices may have contributed. Notably, Gemtuzumab-Ozogamicin has been introduced in AML induction since 2019. Ten patients had a previous treatment with Gemtuzumab-Ozogamicin, all in the OD group. Prophylactic defibrotide was balanced between groups (5 patients each) and did not reduce VOD/SOS in the HARMONY trial [21].

Even so, compared with studies of similar pediatric populations receiving busulfan-based conditioning, our crude incidence exceeds several published series [9,14]. A key explanation lies in diagnostic criteria: unlike prior reports that predominantly applied modified Seattle or Baltimore criteria, we used the revised EBMT criteria. When we reclassified our cohort using modified Seattle criteria, VOD/SOS incidence fell to 26.0%, aligning with those studies. A Danish series showed a similarly sharp gradient across definitions: 44.8% by EBMT, 10.3% by modified Seattle, and 0% by Baltimore criteria [22]. We observed the same pattern (42.0% EBMT; 26.0% Seattle; 8.0% Baltimore). The use of EBMT criteria is, in our view, a strength: these criteria were designed to better capture the pediatric phenotype (earlier onset, frequent anicteric presentation) and have been associated with timelier diagnosis, shorter hospitalizations, and improved response to defibrotide. Refractory thrombocytopenia is the least specific EBMT criterion and was present in 100% and 81.5% of VOD/SOS cases in the OD and QID groups, respectively. AML/MDS and cord blood grafts were more frequent in the OD group and can delay platelet recovery independently of VOD/SOS, possibly overestimating its incidence. However, hepatomegaly and weight gain were nearly as frequent as thrombocytopenia among cases, and such a bias would have favored a higher VOD/SOS incidence with OD, which was not observed.

In multivariable analysis, younger age and thalassemia were independently associated with VOD/SOS. These findings are consistent with prior work: younger children carry a higher VOD/SOS risk, plausibly reflecting hepatic immaturity and endothelial vulnerability [8,9]. Patients with thalassemia have been repeatedly identified as a high-risk group for VOD/SOS, potentially owing to younger age at transplant, iron overload, and chronic inflammation. VOD/SOS is a major determinant of outcomes in this group and a leading cause of transplant-related mortality (TRM) after infection [23]. Elevated pre-HCT ferritin was a significant risk factor for VOD/SOS on univariable analysis but not after adjustment, and several factors reported elsewhere, such as cyclophosphamide-containing regimens, pre-existing liver disease, donor type, and neuroblastoma, did not reach independent significance here. This likely reflects limited statistical power and the heterogeneity of underlying diseases in this single-center cohort.

In our cohort, VOD/SOS incidence did not differ significantly between the OD and QID schedules. This finding is noteworthy given that the OD group was younger (median 5.7 vs. 8.2 years), received Bu/Cy and MUD more often (63.9% vs. 41% and 41.7% vs. 25.6%, respectively), all of which have been associated with an increased risk of hepatic toxicity and VOD/SOS in pediatric HCT. With our sample size, comprehensive multivariable modeling was not feasible; nevertheless, models adjusted for the two variables associated with VOD/SOS in our dataset (age and diagnosis, particularly important in our study since the OD group was younger and all thalassemia patients were in the QID group) did not suggest an independent OD effect, and the odds ratio approached 1. 

The broader literature is mixed but increasingly reassuring regarding schedule choice. In adults, several studies have reported similar or even slightly improved outcomes with OD relative to QID, consistent with earlier comparisons of exposure (AUC), toxicity, and efficacy [15]. Pediatric data are scarcer. Notably, Bartelink et al. observed a higher VOD/SOS incidence with OD IV busulfan plus TDM compared with QID oral busulfan without TDM, yet overall (OS) and event-free survival (EFS) favored the OD group [24]. The design of that study raises interpretive challenges: in the OD arm, early TDM adjusted only once in many patients, risking overexposure, while the comparator relied on oral busulfan, in which underexposure and higher graft failure rates were more common. These factors could artificially depress VOD/SOS risk while worsening efficacy. More recent pediatric series with more contemporary comparators are reassuring: Bazbaz et al. (70 patients; 35 OD/35 QID) found similar EFS and identical VOD/SOS incidence between OD and QID busulfan dosing regimens (criteria not specified) [25]. Taken together with our findings, these data support the view that, when TDM is applied, OD and QID schedules are broadly comparable in pediatric practice.

Our PK analyses showed no difference in AUC_max_ (per-day exposure) or cumulative AUC between OD and QIDtot, and no association of either exposure metric with VOD/SOS. As expected, C_max_ was higher with OD than with QIDtot, reflecting the single daily peak, but this did not translate into increased VOD/SOS risk in our cohort. These observations align with reports that global exposure can be matched between schedules with appropriate TDM [5,24].

The relationship between busulfan exposure and VOD/SOS remains debated. Early IV busulfan experience suggested that higher per-dose AUC could increase VOD/SOS risk, but subsequent studies have not consistently reproduced this association, pointing instead to the role of conditioning partners, especially additional alkylating agents [3,14,26]. Conversely, some analyses have implicated C_max_ as a potential driver: Philippe et al. found that C_max_ was a significant risk factor for VOD/SOS in their pediatric cohort, most of whom received busulfan on a QID schedule [14]. Our data did not support this finding, despite a wider distribution of C_max_ values. One hypothesis is that, in a multiple-dose regimen, C_max_ captures dynamic features of exposure such as repeated peaks, time above threshold, and absorption/elimination kinetics rather than the isolated effect of a single peak.

It is also relevant that 77% of our patients achieved the target cumulative AUC (78–101 mg.h/L), a proportion at the higher end of published experience. Bartelink et al. reported that only 24–32% of patients were within target after the first dose, rising to ~65–70% after TDM-guided adjustment. In our cohort, only 23% fell outside target, and just six patients were potentially overexposed, limiting power to detect exposure–toxicity relationships [3], illustrating the effective PK control achieved in clinical practice. Exposure was not fully comparable between schedules. Mean AUCcum was higher in OD than in QIDtot and QIDallo, with more under-exposure in QIDallo. This may partly reflect the PK approach: each OD day was adjusted on its own full profile, whereas in QID the AUC of one dose was extrapolated to the other three. Despite higher exposure, OD was not associated with more VOD/SOS, and AUCcum was not associated with VOD/SOS risk.

This study has several limitations. First, its single-center, retrospective design, together with the modest sample size (100 transplants; 36 OD and 64 QID) and population heterogeneity, restricts statistical power and may limit the generalizability of the findings. Second, schedule assignment was non-random and correlated with indication and protocol constraints: diagnosis, donor type, conditioning partners, and age differed between groups, introducing confounding that can only be partially addressed in retrospective analyses. Finally, VOD/SOS incidence varied over the 10-year period, with changes in supportive care and diagnostic practices.

The study also has notable strengths. We applied EBMT pediatric criteria and performed a systematic case-by-case review of source records (including daily weights, blood counts, and transfusion logs), yielding complete data on all five EBMT diagnostic criteria for all 100 transplants; this likely improved sensitivity to clinically relevant pediatric VOD/SOS. The TDM program achieved high rates of on-target exposure across both schedules, allowing a comparison of schedule effects independent of gross under- or overexposure. Finally, both schedules were implemented contemporaneously in a single center under routine conditions, which avoids inter-center variability.

## 5. Conclusions

In our study, we did not detect an increased VOD/SOS risk with the OD schedule. Coupled with converging adult data and emerging pediatric series, our findings support the feasibility of OD administration, with respect to VOD/SOS, in children when robust TDM is in place. Beyond safety, an OD schedule carries operational advantages: simplified preparation, reduced drug wastage, fewer infusion events, lower nocturnal nursing workload, and improved patient comfort.

These observations should be confirmed in larger, multicenter cohorts, and, where feasible, in prospective randomized designs to minimize confounding by indication. From a PK–toxicity standpoint, future work should test whether dynamic exposure features provide incremental predictive value beyond cumulative AUC. Stratified analyses in the highest-risk groups (very young children and those with thalassemia or other hepatopathy-prone conditions) are a priority, as these patients stand to gain the most from individualized dosing strategies.

## Figures and Tables

**Table 1:** Patients’ characteristics. CR = complete remission, QIDtot = four-daily-dose schedule, QIDallo = allogeneic QID subgroup, OD = once-daily.

	QIDtot (*n* = 64)	QIDallo (*n* = 39)	OD (*n* = 36)	Total (*n* = 100)	*p*-Value (QIDtot/QIDallo vs. OD)
Age					0.341/0.039
Infant (<12 months)	5 (7.8)	4 (10.2)	4 (11.1)	9	
Toddler (12–23 months)	5 (7.8)	1 (2.6)	6 (16.7)	11	
Preschool child (2–5 years)	16 (25.0)	6 (15.4)	10 (27.8)	26	
School-age child (6–11 years)	24 (37.5)	18 (46.2)	7 (19.4)	31	
Adolescent and young adult (12 years and over)	14 (21.9)	10 (25.6)	9 (25.0)	23	
Sex					0.233/0.378
Female	31 (48.4)	18 (46.2)	13 (36.1)	44	
Male	33 (51.6)	21 (53.8)	23 (63.9)	56	
Underlying disease					
Hematologic malignancies	11 (17.2)	11 (28.2)	18 (50.0)	29	0.001/0.062
Acute lymphoblastic leukemia	8 (12.5)	8 (20.5)	2 (5.5)	10	0.323/0.089
CR1	2	2	2	6	
CR2	3	3	0	3	
Other	3	3	0	1	
Acute myeloid leukemia	2 (3.1)	2 (5.1)	13 (36.1)	15	<0.001/0.001
CR1	1	1	6	7	
CR2	0	0	6	6	
Other	1	1	1	2	
Chronic myeloid leukemia	0 (0)	0 (0)	1 (2.8)	1	0.360/0.480
Other leukemias	0 (0)	0 (0)	2 (5.5)	2	0.127/0.227
Lymphoma	1 (1.6)	1 (2.6)	0 (0)	1	1.0/1.0
Hemoglobinopathies	22 (34.4)	22 (56.4)	6 (16.7)	28	0.067/<0.001
Sickle Cell Anemia	14 (21.9)	14 (35.9)	6 (16.7)	20	0.610/0.072
Thalassemia	8 (12.5)	8 (20.5)	0 (0)	8	0.048/0.005
Solid tumors	25 (39.1)	0 (0)	0 (0)	25	<0.001/NA
Neuroblastoma	21 (32.8)	0 (0)	0 (0)	21	<0.001/NA
Sarcoma	4 (6.3)	0 (0)	0 (0)	4	0.294/NA
Bone marrow failure/myelodysplastic syndrome	2 (3.1)	2 (5.1)	8 (22.2)	10	0.004/0.042
Myelodysplastic syndrome	0 (0)	0 (0)	6 (16.7)	6	0.002/0.01
Bone marrow failure	2 (3.1)	2 (5.1)	2 (5.5)	4	0.617/1.0
Others	4 (6.3)	4 (10.2)	4 (11.1)	7	0.454/1.0
Histiocytosis	1 (1.6)	1 (2.6)	0 (0)	1	1.0/1.0
Inborn errors of metabolism	2 (3.1)	2 (5.1)	1 (2.8)	3	1.0/1.0
Primary immunodeficiencies	1 (1.6)	1 (2.6)	3 (8.3)	4	0.132/0.345
Pre-graft liver abnormalities					0.836/0.647
Yes	30 (46.9)	18 (46.2)	22 (61.1)	52	
Hepatomegaly alone	16 (25.0)	9 (23.1)	15 (41.7)	31	
Other abnormalities (with or without associated hepatomegaly)	14 (21.9)	9 (23.1)	7 (19.4)	21	
No	33 (51.6)	20 (51.3)	14 (38.9)	47	
ND	1 (1.5)	1 (2.5)	0 (0)	1	
Pre-graft bilirubin					0.029/0.002
≤26 µmol/L	49 (76.6)	24 (61.5)	35 (97.3)	84	
>26 µmol/L	12 (18.7)	12 (30.8)	1 (2.7)	13	
ND	3 (4.7)	3 (7.7)	0 (0)	3	
Pre-graft ferritin levels					0.220/0.08
≤1000 µg/L	30 (46.9)	21 (53.8)	20 (55.6)	50	
>1000 µg/L	31 (48.4)	18 (46.2)	12 (33.3)	43	
ND	3 (4.7)	0 (0)	4 (11.1)	7	

**Table 2:** Donor and transplant characteristics. QIDallo = allogeneic QID four-daily-dose schedule subgroup, OD = once-daily, MSD = Matched Sibling Donor, MUD = Matched Unrelated Donor, UCB = umbilical cord blood, Bu = Busulfan, Cy = Cyclophosphamide, Flu = Fludarabine, ATG = Antithymocyte Globulin, ND = Not determined.

	QIDallo (39 Patients)	OD (36 Patients)	Total (75 Patients)	*p*-Value
Type of graft				0.150
Bone marrow	28 (71.7)	22 (61.1)	50 (66.7)	0.341
Peripheral blood	3 (7.7)	4 (11.1)	7 (9.3)	0.704
Cord blood	3 (7.7)	9 (25.0)	12 (16.0)	0.059
Combination	3 (7.7)	1 (2.8)	4 (5.3)	0.616
ND	2 (5.2)	0 (0)	2 (2.7)	0.494
Donor type				0.006
MSD	22 (56.4)	11 (30.5)	33 (44.0)	0.020
MUD	10 (25.6)	15 (41.7)	25 (33.3)	0.035
UCB	3 (7.7)	9 (25.0)	12 (16.0)	0.0502
Haploidentical	2 (5.2)	0 (0)	2 (2,7)	0.494
ND	2 (5.2)	1 (2.8)	3 (4.0)	0.616
Conditioning regimen				0.066
Bu/Cy	16 (41.0)	23 (63.9)	39 (52.0)	0.065
Bu/Flu	7 (17.9)	7 (19.4)	14 (18.7)	1.0
Bu/Flu/Thiotepa	14 (35.9)	4 (11.1)	18 (24.0)	0.01
Others	2 (5.2)	2 (5.6)	4 (5.3)	1.0
Use of ATG				0.604
Yes	29 (74.4)	25 (69.4)	54 (72.0)	
No	10 (25.6)	11 (30.6)	21 (28.0)	

## Data Availability

The data that support the findings of this study are available from the corresponding author upon reasonable request.

## Supplementary Materials

The following supporting information can be downloaded at: https://www.mdpi.com/article/10.3390/jcm15218754/s1, Table S1: First-dose strategy; Table S2: Characteristics of veno-occlusive disease; Table S3: Cumulative AUC according to the administration schedule.

## Abbreviations

The following abbreviations are used in this manuscript:

HCT	Hematopoietic Cell Transplant
PK	Pharmacokinetic
TDM	Therapeutic Drug Monitoring
VOD	Veno-Occlusive Disease
SOS	Sinusoidal Obstruction Syndrome
QID	Four times daily (Quater In Die)
OD	Once Daily
AUC	Area Under the Curve
C_max_	Maximum plasma concentration
AUC_cum_	Cumulative Area Under the Curve
AUC_max_	Highest daily exposure over the 4 treatment days
EBMT	European Society for Blood and Marrow Transplantation
LC-MS/MS	Liquid Chromatography with tandem Mass Spectrometry
COFRAC	French Accreditation Committee
LOD	Limit Of Detection
LOQ	Limit Of Quantification
ATG	Antithymocyte Globulin (Thymoglobulin)
MUD	Matched Unrelated Donor
Bu/Mel	Busulfan/Melphalan
Bu/Cy	Busulfan/Cyclophosphamide
Bu/Flu	Busulfan/Fludarabine
MDS	Myelodysplastic Syndrome
AML	Acute Myeloid Leukemia
ALL	Acute Lymphoblastic Leukemia
CMV	Cytomegalovirus
OS	Overall Survival
EFS	Event-Free Survival
TRM	Transplant-Related Mortality
CI	Confidence Interval
OR	Odds Ratio
UCB	Umbilical Cord Blood
