# Supplementary material for: Simplifying Busulfan Conditioning in Children: Once Daily vs. Four-Times-Daily IV Dosing in Hematopoietic Cell Transplantation
Source: J Clin Med. 2026 Nov 9;15(21):8754. doi: 10.3390/jcm15218754 (PMC13763787; doi:10.3390/jcm15218754)
Supplement: Supplementary file 1 [file jcm-15-08754-s001.zip › jcm-4625308-supplementary.pdf]

Table S1. First-dose strategy

	<9kg	9-16kg	16-23kg	23-34kg	>34kg
QID (dose in mg/kg x4/day)	1	1.2	1.1	0.95	0.8
OD (dose in mg/kg x1/day)	/	4.8	3.6		3.2

Table S2. Characteristics of veno-occlusive disease.

	QID$_{tot}$ (*n*=27)	QID$_{allo}$ (*n*=15)	OD (*n*=15)	p (QID$_{tot}$ / QID$_{allo}$)	Tot (*n*=42)
EBMT criteria, number (percentage)					
Refractory thrombocytopenia	22 (81.5 %)	13 (87%)	15 (100%)	0.142 / 0.483	37 (88%)
Weight gain	23 (85.2%)	14 (93.3%)	13 (86.7 %)	1.00/1.00	36 (86%)
Hepatomegaly	24 (88.9%)	14 (93.3%)	15 (100%)	0.541 / 1.00	39 (93%)
Ascites	16 (59.3%)	11 (73.3%)	11 (73.3%)	0.506 / 1.00	27 (64%)
Elevated bilirubin level	19 (70.3%)	10 (66.7%)	11 (73.3%)	1.00 / 1.00	30 (71%)
Grade					
1	8 (29.6%)	3 (20%)	5 (33%)	0.804 / 0.409	13 (31%)
2	5 (18.5%)	3 (20%)	1 (6.7%)	0.293 / 0.283	6 (14.3%)
3	8 (29.6%)	5 (33.3%)	7 (46.7%)	0.270 / 0.456	15 (35.7%)
4	6 (22.2%)	3 (26.7%)	2 (13.4%)	0.482 / 0.361	8 (19%)

Table S3. Cumulative AUC according to the administration schedule.

	**QID_{tot} (*n*=64)**	**QID_{-allo} (*n*=39)**	**OD (*n*=36)**	**Total (*n*=100)**	**P (QID_{tot}/QID_{allo})**
AUC_{cum} below target, n (%)	13 (20.3)	11 (28.2)	3 (8.3)	16	0.158 / 0.038
AUC_{cum} on-target, n (%)	45 (70.3)	24 (61.5)	27 (75.0)	72	0.651 / 0.228
AUC_{cum} above target, n (%)	2 (3.1)	1 (2.6)	4 (11.1)	6	0.184 / 0.188
ND	4 (6.3)	3 (7.7)	2 (5.6)	6	-
AUC_{cum} (mean)	84.18 ± 9.74	81.68 ± 10.07	89.69 ± 10.77	-	0.016/0.002
